# Illness perception and psychosocial adjustment in young and middle-aged liver transplant recipients during the early postoperative period: the serial mediating roles of perceived social support and quality of life

**DOI:** 10.3389/fpsyg.2026.1869106

**Published:** 2026-06-29

**Authors:** Lixia Fan, Bin Wang, Kai Zhu, Peipei Jia, Yifan Jiang, Xinxin Guo, Yuhan Qiu, Guiyu Xue, Xia Huang

**Affiliations:** 1 School of Nursing, Qingdao University, Qingdao, Shandong, China; 2 Department of Emergency Surgery, The Affiliated Hospital of Qingdao University, Qingdao, Shandong, China; 3 Department of Spinal Surgery, The Affiliated Hospital of Qingdao University, Qingdao, Shandong, China; 4 Department of Sports Medicine, The Affiliated Hospital of Qingdao University, Qingdao, Shandong, China; 5 Organization and Personnel Department, The Affiliated Hospital of Qingdao University, Qingdao, Shandong, China

**Keywords:** illness perception, psychosocial adjustment, quality of life, social support, young and middle-aged liver transplantation

## Abstract

**Objective:**

Psychosocial adjustment is an important component of recovery after liver transplantation, particularly among young and middle-aged recipients during the early postoperative period. Although illness perception has been associated with psychosocial adjustment, the statistical pathways underlying this association in this population remain unclear. This study aimed to examine the association between illness perception and psychosocial adjustment and to determine whether perceived social support and quality of life statistically mediated this association.

**Methods:**

A questionnaire survey was conducted among 216 young and middle-aged liver transplant recipients. The instruments included the Brief Illness Perception Questionnaire (B-IPQ), Perceived Social Support Scale (PSSS), Post-Liver Transplant Quality of Life Questionnaire, and Psychosocial Adjustment to Illness Scale-Self Report (PAIS-SR). Structural equation modeling was used to evaluate the proposed statistical serial mediation model, and the Bootstrap method was adopted to examine the significance of statistical indirect effects.

**Results:**

Illness perception was positively associated with psychosocial adjustment problems (*β* = 0.240, *p* = 0.002). Bootstrap analysis showed significant statistical indirect associations through perceived social support, quality of life, and the sequential pathway of perceived social support and quality of life. The total indirect association accounted for 43.26% of the total association.

**Conclusion:**

Illness perception was associated with poorer psychosocial adjustment among young and middle-aged liver transplant recipients during the early postoperative period, with perceived social support and quality of life serving as statistical mediators. These findings suggest that early postoperative interventions may need to integrate illness-cognition regulation, supportive resource enhancement, and quality-of-life improvement to support psychosocial adjustment.

## 1 Introduction

With continuing advances in surgical techniques, perioperative care, and immunosuppressive therapy, survival after liver transplantation has improved substantially over recent decades (Manzia et al., 2024; Campbell et al., 2026). As a result, the evaluation of transplant outcomes has gradually expanded beyond survival alone to include broader aspects of recovery and long-term functioning (Aberg, 2020). For liver transplant recipients, postoperative recovery involves not only graft function and physical restoration, but also psychological adjustment, social reintegration, and the resumption of everyday roles (Lieber et al., 2022). Psychosocial adjustment is defined as the process by which individuals adjust psychologically and socially to illness-related stress and life changes following a major disease or surgery (Derogatis, 1986). It is an important indicator of postoperative recovery and successful reintegration into everyday life among liver transplant recipients (Vedadi et al., 2023). Therefore, examining psychosocial adjustment after liver transplantation may contribute to a more comprehensive understanding of recovery and inform supportive care (Vedadi et al., 2023; Lieber et al., 2022).

Among liver transplant recipients, young and middle-aged adults account for a large proportion of transplant recipients (Kwong et al., 2024). Compared with older recipients, those of working age often face additional demands during recovery, including return to work, family responsibilities, and social reintegration (Kaplan et al., 2023; Rao et al., 2025). Although liver transplantation has been associated with improved survival and clinical outcomes, recipients may still experience pronounced difficulties in psychological recovery and social reintegration during the early postoperative period, particularly those of working age (Lieber et al., 2022; Rao et al., 2025). Previous studies have suggested that this group is at increased risk of post-transplant psychological problems (Wurm et al., 2022). Approximately 25% report symptoms of depression and anxiety within the first year after transplantation (Biyyala et al., 2023), and these symptoms may be associated with adverse outcomes such as rejection and mortality (Dew et al., 2016; Rogal et al., 2013). In addition, the reported return-to-work rate after liver transplantation is only 28.8% (Fazekas et al., 2021), suggesting persistent challenges in the recovery of social and role functioning. Greater attention to psychosocial adjustment in young and middle-aged liver transplant recipients during the early postoperative period is therefore warranted.

Illness perception refers to patients’ cognitive and emotional representations of their illness, including beliefs about its consequences, duration, controllability, and overall threat (Broadbent et al., 2006). As an important psychological construct, illness perception has been associated with coping responses and subsequent health and recovery outcomes (Hagger et al., 2017). According to the Common-Sense Model of self-regulation, the ways in which individuals interpret and understand their illness may be related to their emotional responses, coping behaviors, and subsequent health outcomes (Leventhal et al., 2016). During the early period after liver transplantation, recipients often face considerable uncertainty and adjustment demands (Lieber et al., 2022). In this context, illness perception may be relevant to psychosocial adjustment. Previous studies in various patient populations have shown that more negative illness perceptions are associated with poorer psychosocial adjustment (Wang et al., 2024). However, this relationship may not be limited to a direct association and may also involve broader psychosocial factors.

One possible pathway involves perceived social support. In liver transplant recipients, social support has been associated with lower psychological distress and better psychosocial adjustment during recovery (Cordoba-Alvarado et al., 2024; De Pasquale et al., 2025). Negative illness perceptions may be associated with lower perceived availability and helpfulness of support, as well as less effective mobilization of support from family members, friends, and healthcare professionals (Hu et al., 2023). In turn, inadequate support may be related to poorer emotional regulation, reduced access to helpful information and guidance, and greater difficulty in adjusting to postoperative demands (Leventhal et al., 2016). Another possible pathway involves quality of life. Quality of life is an important indicator of overall health status, functional recovery, and subjective well-being after transplantation (Cristin et al., 2021). Previous studies have reported a negative association between illness perception and health-related quality of life (Zelikovsky and Nelson, 2021), whereas better quality of life has been linked to lower perceived stress, fewer negative emotional symptoms, and better psychosocial adjustment (Kakemam et al., 2024; Chae et al., 2026). In addition, social support has been associated with quality of life, as adequate support may be related to better emotional experiences, a stronger sense of security, social connectedness, and better quality of life (Chen et al., 2007; Hwang et al., 2021). Taken together, these findings suggest that illness perception may be associated with psychosocial adjustment through perceived social support and quality of life, including their sequential pathway.

Although illness perception, social support, and quality of life have each been associated with psychosocial adjustment, it remains unclear how social support and quality of life are statistically involved in the association between illness perception and psychosocial adjustment among young and middle-aged liver transplant recipients during the early postoperative period (Vedadi et al., 2023; Lieber et al., 2022; Hu et al., 2023). Examining these statistical pathways is of both theoretical and clinical importance, given the substantial demands of psychological recovery and social role reconstruction in this population (Kaplan et al., 2023; Lieber et al., 2022). This study therefore aimed to examine the relationship between illness perception and psychosocial adjustment and to assess whether social support and quality of life statistically mediated this relationship, both independently and sequentially.

## 2 Literature review and research hypotheses

### 2.1 The association between illness perception and psychosocial adjustment

Although young and middle-aged liver transplant recipients generally face dual demands of treatment-related recovery and role reconstruction during the early postoperative period, individual differences in psychosocial adjustment remain evident (Lieber et al., 2022). This suggests that, under similar treatment conditions, patients’ subjective appraisal of illness may be associated with postoperative psychosocial adjustment (Dempster et al., 2015). Illness perception reflects patients’ understanding of the nature, course, consequences, and controllability of illness, and may be related to their appraisal of illness-related threats and subsequent coping responses (Hagger et al., 2017). Studies across different patient populations have consistently shown that more negative illness perceptions are associated with poorer psychosocial adjustment (Quiles Marcos et al., 2007; Wang et al., 2024), with similar findings reported among liver transplant recipients (Martín-Rodríguez et al., 2012). More positive illness perceptions have been associated with greater recovery confidence, perceived control, and active coping, whereas more negative illness perceptions have been associated with greater perceived threat, uncontrollability, anxiety, helplessness, and psychosocial adjustment difficulties (Marcil et al., 2023; O’Connor et al., 2023).

Therefore, Hypothesis H1 is proposed: More negative illness perceptions are associated with poorer psychosocial adjustment among young and middle-aged liver transplant recipients during the early postoperative period.

### 2.2 The statistical mediating effect of social support

Perceived social support is a protective factor associated with better postoperative psychosocial adjustment (Li et al., 2024; Zell and Stockus, 2025). It refers to individuals’ perception of emotional, informational, and instrumental assistance from family members, friends, and significant others (Tjin et al., 2022). For young and middle-aged liver transplant recipients, perceived social support may be associated with access to external resources for managing long-term medication use, follow-up monitoring, and role recovery after transplantation (Zhang et al., 2022). According to the stress-buffering model, social support has been associated with lower stress-related difficulties and may be related to patients’ appraisal of illness- and treatment-related stressors and adaptive coping responses (Acoba, 2024; Cohen and Wills, 1985). Empirical studies have shown that more negative illness perception is associated with lower perceived social support, whereas higher perceived social support is linked to more active coping and better psychological and social functioning (Al-Zaru and Al-Dwairi, 2023; Segal et al., 2021). Among liver transplant recipients, perceived social support may be associated with lower treatment uncertainty, lower recovery-related stress, and better psychosocial adjustment (Ordin and Karayurt, 2016). Therefore, perceived social support may serve as a statistical mediator in the relationship between illness perception and psychosocial adjustment.

Hypothesis H2 is therefore proposed: Perceived social support serves as a statistical mediator in the association between illness perception and psychosocial adjustment.

### 2.3 The statistical mediating effect of quality of life

Quality of life is a key indicator for evaluating overall postoperative recovery (Aberg, 2020). As a multidimensional construct, it reflects individuals’ perceptions of their health status, functional ability, psychological well-being, social relationships, and daily functioning (Skevington et al., 2004). Patients with more negative illness perceptions may perceive disease consequences, treatment burden, and recovery as more threatening and may be more likely to report poorer health-related quality of life (Wang et al., 2023; Knowles et al., 2023). Insufficient perceived social support may be associated with poorer quality of life, greater helplessness, and less effective coping with illness- and recovery-related challenges (Katz et al., 2016; Drageset, 2021). Lower quality of life has also been associated with greater psychological burden, restricted functional recovery, reduced social participation, and poorer psychosocial adjustment (Aberg, 2020; Dunn et al., 2020; Cordoba-Alvarado et al., 2024). These findings suggest that quality of life may serve as a statistical mediator in the associations among illness perception, perceived social support, and psychosocial adjustment, and that examining these statistical pathways may provide useful information for supportive interventions during the early postoperative period.

Hypothesis H3 is therefore proposed: Quality of life serves as a statistical mediator in the association between illness perception and psychosocial adjustment.

### 2.4 The sequential statistical mediating role of social support and quality of life

**Figure 1:**
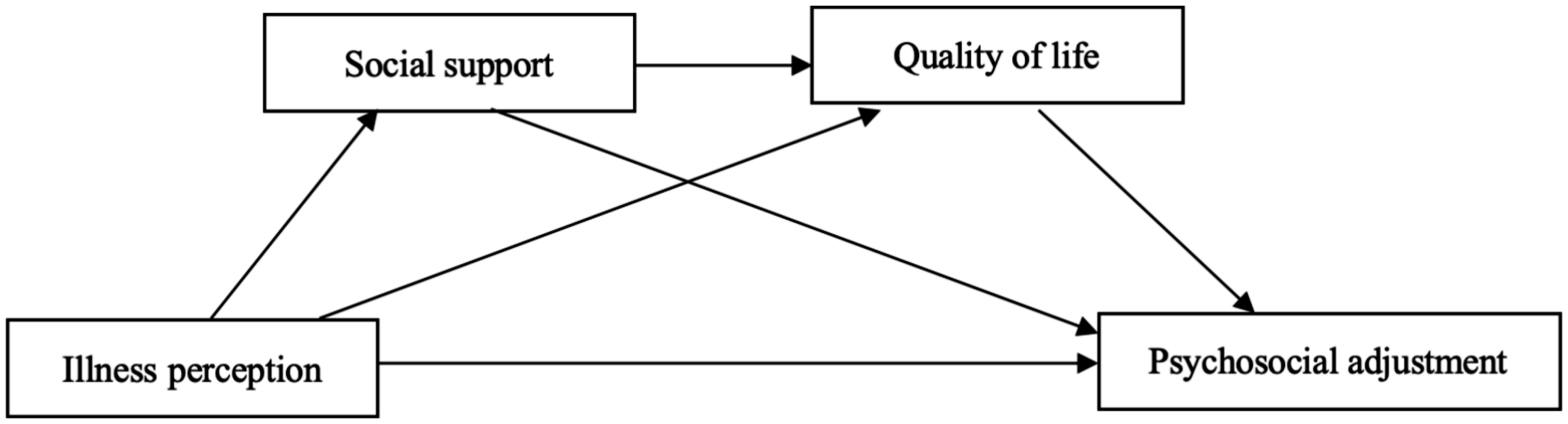
Proposed serial mediation model.

Although previous studies have examined the separate associations of illness perception, perceived social support, and quality of life with psychosocial adjustment in liver transplant recipients, no empirical study has integrated these factors into a statistical model of psychosocial adjustment among young and middle-aged recipients during the early postoperative period. Given the treatment-related stress, functional recovery tasks, and role reconstruction demands faced by this population, examining these statistical pathways may provide useful information for understanding early postoperative recovery and supportive care. Therefore, this study aimed to examine the association between illness perception and psychosocial adjustment and to assess whether perceived social support and quality of life statistically mediated this association, both independently and sequentially. The research hypotheses were proposed as follows (Figure 1):

*H1*: More negative illness perceptions are positively associated with poorer psychosocial adjustment among young and middle-aged liver transplant recipients during the early postoperative period.

*H2*: Perceived social support serves as a statistical mediator in the association between illness perception and poorer psychosocial adjustment.

*H3*: Quality of life serves as a statistical mediator in the association between illness perception and poorer psychosocial adjustment.

*H4*: Perceived social support and quality of life serve as sequential statistical mediators in the association between illness perception and poorer psychosocial adjustment.

## 3 Method

### 3.1 Study design

A cross-sectional questionnaire-based study was conducted between July 2025 and February 2026.

### 3.2 Study setting and participants

We used convenience sampling to recruit young and middle-aged liver transplant recipients during the early postoperative period from the Department of Organ Transplantation at a tertiary hospital in Shandong Province, China. A total of 220 questionnaires were distributed to eligible hospitalized patients. After data collection, all questionnaires were checked for completeness and response quality. Four questionnaires were excluded because of incomplete responses or an excessively short completion time. Finally, 216 valid responses were included in the final analysis, yielding a valid questionnaires rate of 98.18%.

Participants were included if they: (1) had undergone their first liver transplantation within the past 6 months; (2) were aged 18–59 years; (3) were conscious and able to communicate normally; and (4) provided informed consent. Participants were excluded if they: (1) had a history of psychiatric disorders or cognitive impairment; (2) had undergone transplantation of another organ; or (3) had severe cardiac, pulmonary, or renal dysfunction that prevented participation.

The sample size was estimated using the formula *n = (Z*₁-*α*/₂ × *σ* /*δ)*^2^, where *n* represents the required sample size, *Z*₁-*α*/₂ represents the standard normal deviate corresponding to a two-sided significance level, *σ* represents the standard deviation of psychosocial adjustment scores among organ transplant recipients, and *δ* represents the allowable error. In this study, the significance level was set at *α* = 0.05, corresponding to *Z*₁-*α*/₂ = 1.96. Based on previous literature (Hu et al., 2023), *σ* was 16.39. The allowable error was set at 0.15 times the standard deviation; therefore, *δ* = 2.46. The calculated minimum sample size was 171 participants. After accounting for an anticipated 20% invalid or incomplete response rate, the minimum required sample size was 214 participants. The final sample size of 216 exceeded the commonly used reference value of 200 for relatively simple SEM models and was therefore considered acceptable for estimating the path model and conducting Bootstrap mediation analysis (Kline, 2023).

### 3.3 Measures

#### 3.3.1 Psychosocial adjustment

Psychosocial adjustment was assessed using the Psychosocial Adjustment to Illness Scale–Self-Report (Derogatis, 1986). The Chinese version was translated and adapted by Yao (2013). The PAIS-SR includes 44 items across seven dimensions, rated on a 4-point Likert scale. The total score ranges from 0 to 132, with higher scores indicating more severe psychosocial adjustment problems, that is, poorer psychosocial adjustment. In this study, Cronbach’s *α* was 0.872.

#### 3.3.2 Quality of life

Quality of life was measured using the Chinese version of the Post-Liver Transplant Quality of Life Questionnaire (PLTQ) (Younossi et al., 1999), which was translated and adapted by Peng et al. (2015). The PLTQ contains 28 items across six dimensions and uses a 7-point Likert scale. The total score ranges from 28 to 196, with higher scores indicating better quality of life. In this study, Cronbach’s *α* was 0.939.

#### 3.3.3 Social support

Social support was measured using the Perceived Social Support Scale (PSSS) (Zimet et al., 1990). The Chinese version was revised by (Jiang et al., 1996). It comprises 12 items across 3 dimensions and is rated on a 7-point Likert scale. The total score ranges from 12 to 84, with higher scores indicating a higher level of perceived social support. In this study, Cronbach’s *α* was 0.920.

#### 3.3.4 Illness perception

Illness perception was assessed using the Brief Illness Perception Questionnaire (B-IPQ) (Broadbent et al., 2006). The Chinese version was translated and cross-culturally adapted by Mei et al. (2015). The B-IPQ evaluates patients’ cognitive and emotional representations of illness. It includes eight scored items, each rated from 0 to 10, with a total score ranging from 0 to 80. Higher total scores indicate more negative illness perceptions. In this study, Cronbach’s *α* was 0.770.

### 3.4 Demographic and clinical characteristics

Sociodemographic and clinical variables included age, sex, educational level, occupation, marital status, fertility status, place of residence, monthly per capita household income, medical payment method, comorbid chronic disease, work status after liver transplantation, etiology of liver transplantation, type of liver transplantation, and time since transplantation.

### 3.5 Covariates

Age and time since liver transplantation were specified as observed exogenous variables, and their effects on psychosocial adjustment problems were controlled for when estimating the direct and indirect effects of the serial mediation model. Age was categorized into two groups: 18–40 years and 41–59 years. Time since liver transplantation was divided into three groups: <1 month, 1–3 months, and more than 3 and up to 6 months.

### 3.6 Data collection procedures

Prior to data collection, all investigators received standardized training on the study purpose, eligibility criteria, and questionnaire administration procedures to ensure consistency throughout the survey process. Participants were recruited strictly in accordance with the predefined inclusion and exclusion criteria. For those with lower educational attainment or limited comprehension, face-to-face assistance was provided using standardized instructions to minimize information bias. All completed questionnaires were reviewed immediately on site for completeness and missing responses. After collection, all questionnaires were independently checked by two researchers before data entry to ensure accuracy and completeness.

### 3.7 Ethics statement

This study was reviewed and approved by the hospital ethics committee (approval No. QYFY WZLL 50025). Written informed consent was obtained from all participants prior to data collection.

### 3.8 Statistical analysis

Data were analyzed using SPSS version 27.0 and AMOS version 26.0. Common method bias was examined using Harman’s single-factor test. All measurement items were entered into an exploratory factor analysis, and a variance contribution of less than 40% for the first unrotated factor was considered to indicate the absence of serious common method bias (Podsakoff et al., 2003).

Descriptive statistics were used to summarize participants’ demographic and clinical characteristics and the main study variables. Normally distributed continuous variables were expressed as mean and standard deviation, whereas categorical variables were described using frequencies and percentages. The normality of psychosocial adjustment scores was assessed using the Shapiro–Wilk test and Q-Q plots, and homogeneity of variance was examined using Levene’s test. When the assumptions of normality and homogeneity of variance were met, independent-samples *t* tests or one-way ANOVA were used. Pearson correlation analysis was conducted to examine the associations among illness perception, perceived social support, quality of life, and psychosocial adjustment problems.

Structural equation modeling was used to evaluate the proposed serial mediation model. In this study, latent variables were constructed using dimension scores or item parcel scores as observed indicators. The measurement model was first tested to examine whether the observed indicators adequately represented the latent constructs. The structural model was then used to examine the direct and indirect pathways among the latent variables, with illness perception as the independent variable, perceived social support and quality of life as mediators, and psychosocial adjustment problems as the dependent variable. To reduce the potential influence of confounding factors, an adjusted structural equation model was further conducted by including age and time since liver transplantation as key covariates. Age and time since liver transplantation were specified as observed exogenous variables, and their effects on psychosocial adjustment problems were controlled for when estimating the direct and indirect effects of the serial mediation model. The covariates were also allowed to correlate with other exogenous variables in the model. Model fit was evaluated using the chi-square/degrees of freedom ratio (*χ*^2^/*df*), root mean square error of approximation (RMSEA), standardized root mean square residual (SRMR), comparative fit index (CFI), and Tucker–Lewis index (TLI). The significance of the statistical indirect effects was examined using the bias-corrected bootstrap method with 5,000 resamples. Statistical mediation was considered significant when the 95% confidence interval did not include zero. All tests were two-tailed, and *p* < 0.05 was considered statistically significant.

## 4 Results

### 4.1 Assessment of common method bias

Harman’s single-factor test identified 23 factors with eigenvalues greater than 1, and the first unrotated factor explained 21.718% of the total variance, suggesting that common method bias was unlikely to be a serious concern in this study.

### 4.2 Measurement model

Confirmatory factor analysis was conducted to evaluate the measurement model. The results showed that the measurement model had a generally acceptable fit to the data: *χ*^2^/*df* = 2.114, SRMR = 0.062, RMSEA = 0.072, and CFI = 0.895. Although the CFI was slightly below the conventional threshold of 0.90, the *χ*^2^/*df*, SRMR, and RMSEA values were within acceptable ranges, suggesting that the overall measurement model was acceptable. The standardized factor loadings ranged from 0.576 to 0.698 for illness perception, from 0.757 to 0.815 for perceived social support, from 0.797 to 0.869 for quality of life, and from 0.588 to 0.687 for psychosocial adjustment. CR values for illness perception, perceived social support, quality of life, and psychosocial adjustment were 0.854, 0.837, 0.934, and 0.835, respectively, all exceeding 0.70. AVE values were 0.423, 0.632, 0.704, and 0.420, respectively. Although the AVE values for illness perception and psychosocial adjustment were slightly below 0.50, their CR values were above 0.80, and the standardized factor loadings were acceptable; therefore, the convergent validity of the measurement model was considered generally acceptable. Discriminant validity was examined by comparing the square roots of AVE with the correlations among latent constructs. The square roots of AVE for illness perception, perceived social support, quality of life, and psychosocial adjustment were 0.651, 0.795, 0.839, and 0.648, respectively. These values were greater than the absolute values of the correlations between the corresponding constructs and other latent variables, indicating acceptable discriminant validity among the four latent constructs.

### 4.3 Psychosocial adjustment across participant characteristics

Psychosocial adjustment scores differed significantly by age, educational level, work status, place of residence, parental status, monthly per capita household income, comorbid chronic disease, and time since transplantation (all *p* < 0.05). No significant differences were observed by sex, marital status, occupation, medical payment method, etiology of liver transplantation, or type of liver transplantation. Detailed results are presented in Table 1.

**Table 1:** Comparison of early postoperative psychosocial adjustment scores among young and middle-aged liver transplant recipients with different characteristics (*n* = 216).

**Variable**	***n***	**Psychosocial adjustment** **(*M* ± *SD*)**	***t/F***	***p value***	**Variable**	***n***	**Psychosocial adjustment** **(*M* ± *SD*)**	***t/F***	***p value***
Age (years)			−2.12	<0.05	Monthly per capita household income			2.95^a^	<0.05
18–40	28	70.93 ± 14.15			< 2,000 RMB	10	86.10 ± 14.89		
41–59	188	77.28 ± 14.92			2,000–4,000 RMB	68	79.04 ± 16.28		
Educational level			11.85^a^	<0.001	4,001–6,000 RMB	101	74.40 ± 14.08		
Illiterate	6	102.33 ± 8.07			≥ 6,000 RMB	37	74.73 ± 13.46		
Primary school	12	96.58 ± 11.84			Chronic disease			−3.91	<0.001
Junior middle school	52	76.90 ± 15.31			Yes	156	74.07 ± 14.33		
High school/secondary technical school	52	75.81 ± 14.11			No	60	82.60 ± 14.85		
Junior college	48	71.50 ± 11.33			Time since liver transplantation			19.46^a^	<0.001
Bachelor’s degree or above	46	73.24 ± 12.86			< 1 month	40	85.13 ± 14.75		
Work status			−2.18	<0.05	1–3 months	90	78.99 ± 14.13		
Returned to work	23	70.09 ± 15.11			> 3 to 6 months	86	69.78 ± 12.99		
Not yet returned to work	193	77.22 ± 14.78			Fertility status			3.51^a^	<0.05
Place of residence			−2.37	< 0.05	1 child	16	85.81 ± 15.11		
Urban	179	75.37 ± 14.27			2 children	153	76.47 ± 15.01		
Rural	37	81.70 ± 17.11			3 children	38	71.84 ± 13.80		
					4 or more children	9	79.11 ± 11.29		

ᵃ*F* value.

### 4.4 Descriptive statistics of the main variables

Among the 216 participants, the mean scores were 45.14 ± 5.64 for illness perception, 34.09 ± 6.11 for social support, 137.83 ± 18.40 for quality of life, and 76.46 ± 14.95 for psychosocial adjustment problems. Scores for each dimension and mean item scores are presented in Table 2.

**Table 2:** Scores of B-IPQ, PSSS, PLTQ, and PAIS-SR in young and middle-aged liver transplant recipients during the early postoperative period.

**Instrument**	**Total score (*M* ± *SD*)**	**Mean item score *(M ± SD)***
B-IPQ	45.14 ± 5.64	5.64 ± 0.70
PSSS	34.09 ± 6.11	2.84 ± 0.51
PLTQ	137.83 ± 18.40	4.93 ± 0.66
PAIS-SR	76.46 ± 14.95	1.74 ± 0.34

B-IPQ = Brief Illness Perception Questionnaire; PSSS = Perceived Social Support Scale; PLTQ = Chinese version of the Post-Liver Transplant Quality of Life Questionnaire; PAIS-SR = Psychosocial Adjustment to Illness Scale–Self Report.

### 4.5 Model fit and modification

The initial hypothesized model showed a partially acceptable but not ideal fit, with comparative fit indices slightly below the recommended threshold. Therefore, the model was further modified based on modification indices and theoretical considerations. Two residual covariance paths were added between e18 and e21 and between e22 and e23. These indicators belonged to the same latent construct of illness perception and may have shared measurement variance not fully explained by the latent variable. No additional structural paths were added. After modification, the model fit improved and reached an acceptable level, indicating that the modified model was suitable for subsequent path analysis and mediation effect testing. The fit indices of the initial and modified models are presented in Table 3.

**Table 3:** Fit indices of the initial and modified structural equation models.

**Model**	***χ*** ^**2**^ **/*df***	**RMSEA**	**SRMR**	**CFI**	**TLI**
Initial model	2.001	0.068	0.064	0.890	0.878
Modified model	1.639	0.055	0.060	0.931	0.922

### 4.6 Correlations among the main study variables

Pearson correlation analysis showed that psychosocial adjustment problems were positively correlated with illness perception and negatively correlated with social support and quality of life (all *p* < 0.01). Illness perception was negatively correlated with perceived social support and quality of life, whereas perceived social support was positively correlated with quality of life (all *p* < 0.01). Detailed correlation coefficients are presented in Table 4.

**Table 4:** Pearson correlations among the main study variables in young and middle-aged liver transplant recipients during the early postoperative period.

**Variable**	**Illness perception**	**Social support**	**Quality of life**	**Psychosocial adjustment**
Illness perception	1	—	—	—
Social support	−0.343^b^	1	—	—
Quality of life	−0.409^b^	0.434^b^	1	—
Psychosocial adjustment	0.368^b^	−0.361^b^	−0.419^b^	1

ᵇ*p* < 0.01. Values in the upper triangle are not shown.

### 4.7 Serial mediation analysis

**Figure 2:**
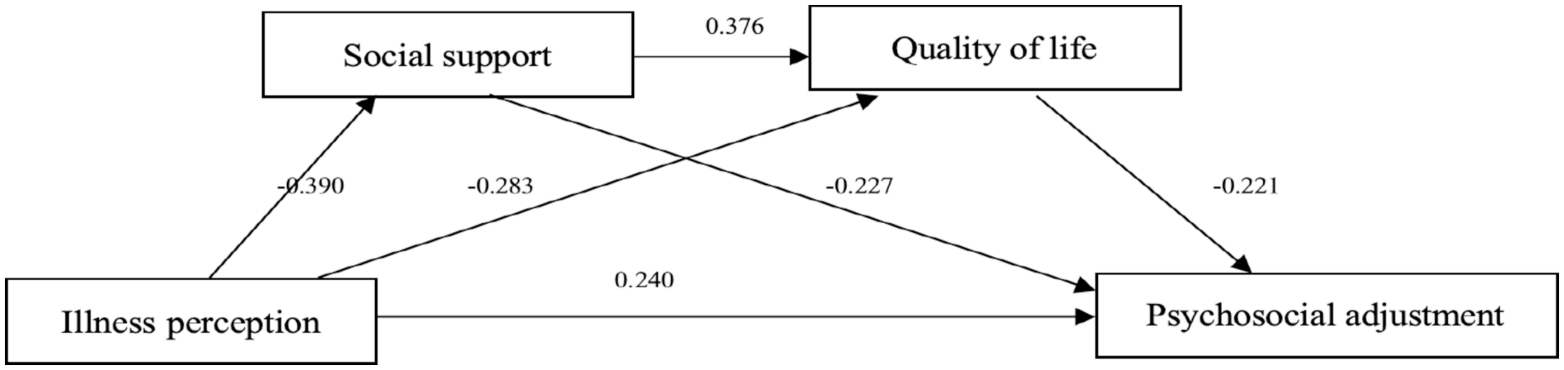
Serial mediation model of perceived social support and quality of life in the relationship between illness perception and psychosocial adjustment among young and middle-aged liver transplant recipients during the early postoperative period.

A structural equation model was constructed with illness perception as the independent variable, social support and quality of life as statistical mediators, and psychosocial adjustment problems as the dependent variable. The initial model was estimated using maximum likelihood, and a modified model was obtained based on the modification indices. The standardized model is shown in Figure 2.

The final adjusted structural equation model showed statistically significant associations among illness perception, perceived social support, quality of life, and psychosocial adjustment problems. After controlling for age and time since liver transplantation, illness perception was negatively associated with perceived social support (*β* = −0.390, *p* < 0.001) and quality of life (*β* = −0.283, *p* < 0.001), and was positively associated with psychosocial adjustment problems (*β* = 0.240, *p* = 0.002). Perceived social support was positively associated with quality of life (*β* = 0.376, *p* < 0.001) and negatively associated with psychosocial adjustment problems (*β* = −0.227, *p* = 0.007). Quality of life was also negatively associated with psychosocial adjustment problems (*β* = −0.221, *p* = 0.006). The standardized and unstandardized path coefficients are reported in Table 5.

**Table 5:** Standardized and unstandardized path coefficients of the structural equation model.

Outcome variable	Predictor variable	***β***	***b***	**SE**	**CR**	***P***
Social support	Illness perception	−0.390	−1.057	0.222	−4.771	< 0.001
Quality of life	Illness perception	−0.283	−0.830	0.223	−3.715	< 0.001
Quality of life	Social support	0.376	0.407	0.085	4.758	< 0.001
Psychosocial adjustment	Social support	−0.227	−0.255	0.094	−2.720	0.007
Psychosocial adjustment	Quality of life	−0.221	−0.229	0.083	−2.747	0.006
Psychosocial adjustment	Illness perception	0.240	0.731	0.241	3.038	0.002

β = standardized coefficient; b = unstandardized coefficient; SE = standard error; CR = critical ratio; P = probability value.

Bootstrap analysis indicated a significant statistical mediation pathway involving perceived social support and quality of life in the association between illness perception and psychosocial adjustment problems. The total indirect effect accounted for 43.26% of the total effect. Specifically, the indirect effect through perceived social support alone accounted for 20.80%, the indirect effect through quality of life alone accounted for 14.66%, and the sequential indirect effect through perceived social support and quality of life accounted for 7.57%. The detailed bootstrap mediation results are shown in Table 6.

**Table 6:** Serial mediation effects of illness perception on psychosocial adjustment problems through social support and quality of life.

Path	***β***	**SE**	**95% CI**	***P***	**Proportion of total effect** **(%)**
Indirect effect 1: Illness perception → Perceived social support → Psychosocial adjustment	0.088	0.040	0.025–0.188	0.005	20.80%
Indirect effect 2: Illness perception → Quality of life → Psychosocial adjustment	0.062	0.029	0.018–0.135	0.003	14.66%
Indirect effect 3: Illness perception → Perceived social support → Quality of life → Psychosocial adjustment	0.032	0.015	0.011–0.075	0.002	7.57%
Total indirect effect	0.183	0.044	0.107–0.284	<0.001	43.26%
Direct effect	0.240	0.073	0.095–0.390	<0.001	56.74%
Total effect	0.423	0.066	0.294–0.549	<0.001	100.00%

The proportions were calculated based on unrounded estimates. Minor discrepancies in summed percentages may be due to rounding.

## 5 Discussion

This study used a serial mediation model to examine how illness perception was associated with psychosocial adjustment problems among young and middle-aged liver transplant recipients during the early postoperative period. The findings showed that illness perception was associated with psychosocial adjustment problems through statistical direct and indirect pathways involving perceived social support and quality of life. These results suggest that psychosocial adjustment problems in this population may be related not only to illness perception, but also to supportive resources and recovery-related experiences (Hagger et al., 2017; Vedadi et al., 2023; Hu et al., 2023).

### 5.1 Psychosocial adjustment across participant characteristics

Early postoperative psychosocial adjustment scores differed by age, educational level, work status after liver transplantation, place of residence, monthly per capita household income, comorbid chronic disease, time since transplantation, and fertility status, but not by sex, occupation, marital status, medical payment method, etiology of liver transplantation, or type of liver transplantation. The nonsignificant finding for marital status may be related to the predominance of married participants and the imbalanced distribution across marital status groups. Moreover, marital status may not fully represent the quality, stability, or availability of actual support. A previous study on liver transplant recipients and their family caregivers showed that patients’ psychological experiences were closely related to perceived social support, dependency relationships, and caregiver-related factors, suggesting that actual support networks may better reflect patients’ supportive resources than marital status alone (Cipolletta et al., 2019). The nonsignificant findings for sex and occupation may indicate that, during the early postoperative period, recipients with different sex and occupational backgrounds may face similar recovery-related demands (Cordoba-Alvarado et al., 2024; Campillo Amo et al., 2024). In this study, etiology of liver transplantation and type of liver transplantation were not significantly associated with psychosocial adjustment scores. Although patients with different etiologies may differ in their preoperative illness experiences and psychological burden, they may face similar treatment management and recovery-related demands during the early postoperative period (Shizuku et al., 2021). The nonsignificant finding for type of liver transplantation may be related to the fact that surgical approaches are often selected according to medical factors (Pollok et al., 2023). In addition, medical payment method may not fully capture patients’ actual financial toxicity or ongoing treatment burden, such as out-of-pocket expenses, long-term medication costs, and loss of work productivity (Ufere et al., 2024). These nonsignificant findings should be further examined in future studies with larger samples.

### 5.2 The association between illness perception and psychosocial adjustment problems

Illness perception was associated with psychosocial adjustment problems through a direct statistical pathway, indicating that more negative illness perceptions were associated with poorer psychosocial adjustment. This finding is consistent with previous research and supports the view that patients’ cognitive and emotional representations of illness may be relevant to post-transplant psychosocial adjustment (Broadbent et al., 2006; Hu et al., 2023). During the early postoperative period, young and middle-aged recipients often face substantial uncertainty related to recovery, treatment burden, and role resumption. When illness is perceived as highly threatening or difficult to control, patients may be more likely to experience negative emotional responses and adopt less adaptive coping strategies, which may be associated with poorer psychosocial adjustment (Leventhal et al., 2016; Hagger et al., 2017). Clinically, healthcare professionals may consider assessing illness perception during early postoperative follow-up and identifying recipients with more negative illness perceptions. For these patients, individualized education, brief cognitive counseling, and teach-back approaches may be used to support a more realistic understanding of recovery, long-term immunosuppressive therapy, complication prevention, symptom self-monitoring, and daily self-management after transplantation (Talevski et al., 2020; Kim et al., 2024).

### 5.3 The statistical mediating role of perceived social support

Perceived social support served as a partial statistical mediator in the association between illness perception and psychosocial adjustment problems. This finding is in line with previous evidence and may be understood in relation to the buffering role of social support (Cohen and Wills, 1985; Hu et al., 2023). More negative illness perceptions may be associated with lower perceived availability and helpfulness of support, as well as less effective mobilization of supportive resources from family members, friends, and healthcare professionals (Hu et al., 2023; Leventhal et al., 2016). Such limited support may be related to poorer emotional regulation, reduced access to helpful information and guidance, and greater difficulty in adjusting to postoperative demands (Castellano-Santana et al., 2025). This statistical pathway may be particularly relevant for young and middle-aged recipients, who often need to balance recovery with family and occupational responsibilities. Clinically, healthcare professionals may consider assessing perceived social support during early follow-up and identifying recipients with limited support. For these patients, family caregivers may be involved in discharge education and follow-up counseling, and nurse-led telephone or online follow-up, peer support, family communication guidance, or psychological referral may be considered to provide emotional, informational, and practical support during recovery (Zhang et al., 2023; Kim et al., 2024; Lerret et al., 2022; Cipolletta et al., 2019).

### 5.4 The statistical mediating role of quality of life

Quality of life served as a partial statistical mediator in the association between illness perception and psychosocial adjustment problems. This finding suggests that illness perception may be associated with psychosocial adjustment problems, at least in part, through a statistical pathway involving patients’ broader evaluations of their physical, psychological, and social functioning. In the early postoperative period, ongoing symptoms, medication-related adverse effects, repeated follow-up, and disruption of daily roles may all be associated with quality of life (Taher et al., 2021; Onghena et al., 2016; Chen et al., 2012). When illness perceptions are more negative, patients may be more likely to evaluate their recovery and functioning unfavorably, which may be associated with more psychosocial adjustment problems. Clinically, healthcare professionals may consider assessing quality of life during early follow-up and identifying recipients with poor recovery experiences. For these patients, postoperative supportive care may include symptom management, monitoring of medication-related adverse effects, assessment of sleep problems and fatigue, nutritional guidance, individualized rehabilitation, and counseling on return to family, social, and work roles (Kaplan et al., 2023; Lieber et al., 2022; Pollok et al., 2023).

### 5.5 The sequential statistical mediating role of perceived social support and quality of life

Notably, this study showed a significant sequential statistical mediation pathway involving perceived social support and quality of life in the association between illness perception and psychosocial adjustment problems. This suggests that more negative illness perceptions may be associated with lower perceived social support and less effective mobilization of supportive resources, which may also be related to poorer quality of life and more psychosocial adjustment problems (Vedadi et al., 2023; Hu et al., 2023; Leventhal et al., 2016). Perceived social support and quality of life may not operate merely as parallel factors; rather, they may be statistically connected in a sequential pathway. Perceived social support reflects the external resources available to patients during illness-related psychosocial adjustment (Garcia et al., 2018), whereas quality of life reflects patients’ broader evaluations of their physical, psychological, and social functioning during recovery (Greco et al., 2023). This extends previous work by suggesting that supportive resources may be relevant not only in their own right but also through their association with quality of life. Clinically, this sequential pathway suggests that illness perception, perceived social support, and quality of life may be incorporated into routine early postoperative follow-up rather than assessed separately or only once. Healthcare professionals may consider using a brief follow-up checklist to regularly review patients’ illness perception, available support, symptom experience, functional recovery, and quality of life. For recipients reporting persistent negative illness perceptions, limited support, or poor recovery experiences, nurses may document changes over time and coordinate timely support from transplant physicians, family caregivers, rehabilitation staff, or mental health professionals when needed (van Zanten et al., 2025; Lerret et al., 2022; Kim et al., 2024).

## 6 Strengths and limitations

This study has several strengths. First, it focused on a relatively unique and under-researched population: young and middle-aged liver transplant recipients during the early postoperative period. Second, by incorporating illness perception, perceived social support, quality of life, and psychosocial adjustment problems into a single analytical framework, this study provided a more systematic understanding of factors associated with psychosocial adjustment after liver transplantation. Third, this study employed a serial mediation model, which examined not only the direct association among variables but also the potential statistical indirect pathways through which illness perception was associated with psychosocial adjustment problems. These findings provide preliminary evidence for understanding early postoperative psychosocial adjustment difficulties among young and middle-aged liver transplant recipients.

This study has several limitations. First, it employed a cross-sectional design; therefore, causal inferences cannot be drawn. Although the results suggest significant associations among illness perception, perceived social support, quality of life, and psychosocial adjustment problems, the temporal sequence and direction of these associations remain unclear and require further validation through longitudinal studies. Reverse causality also cannot be ruled out; for example, recipients with poorer psychosocial adjustment may report more negative illness perceptions. Future longitudinal studies, such as cross-lagged panel designs, are needed to clarify the directionality and potential reciprocal associations among these variables. Second, this study used convenience sampling and was conducted at a single tertiary hospital in Shandong Province. As a result, the representativeness of the sample may be limited, which may limit the generalizability of the findings. Future multicenter studies involving samples from different regions are needed to further validate these findings. Third, all variables were measured using self-report questionnaires, which may introduce reporting bias. Although the test for common method bias indicated that this was not a serious concern, the possibility of bias arising from self-reported data cannot be ruled out. Fourth, although this study identified several significant statistical mediating pathways, it did not include other factors that may be associated with psychosocial adjustment, such as symptom burden, self-efficacy, and family functioning. These factors should be considered in future research. Finally, this study did not conduct multi-group analyses stratified by sex, age, or educational level. Therefore, it remains unclear whether the associations among illness perception, perceived social support, quality of life, and psychosocial adjustment problems differ across demographic subgroups. Future studies with larger and more diverse samples are needed to examine potential subgroup differences and to assess the stability of the proposed statistical model across different populations.

## 7 Conclusion

In summary, illness perception was associated with poorer psychosocial adjustment in young and middle-aged liver transplant recipients during the early postoperative period through statistically significant direct and indirect pathways involving perceived social support and quality of life. Perceived social support and quality of life were also involved in a significant sequential statistical pathway linking illness perception to psychosocial adjustment problems. These findings suggest that integrated postoperative supportive care may need to consider illness perception, supportive resources, and quality of life to better support psychosocial adjustment in this population.

## Data Availability

The raw data supporting the conclusions of this article will be made available by the authors, without undue reservation.
